# Bio-banding in football: a scoping review with evidence gap map

**DOI:** 10.3389/fpsyg.2026.1960364

**Published:** 2026-09-16

**Authors:** Jihang Zhang, Rui Bao, Liuyi Wang, Shihan Zhuge, Pengyu Pan, Da An, Junmao Chen, Haoyang Yu, Xinjie Zhuo

**Affiliations:** 1 College of Physical Education and Health, East China Normal University, Shanghai, China; 2 School of Sport, Exercise and Health Sciences, Loughborough University, Loughborough, United Kingdom; 3 School of Physical Education, Shenzhen University, Shenzhen, China; 4 Department of Health and Physical Education, The Education University of Hong Kong, Taipo, Hong Kong SAR, China; 5 Institute of Exercise Training and Sports Informatics, German Sport University Cologne, Cologne, Germany; 6 School of Physical Education, Nanchang University, Nanchang, China; 7 College of Physical Education, Inner Mongolian Minzu University, Tongliao, China; 8 College of Physical Education and Sports, Beijing Normal University, Beijing, China

**Keywords:** grouping strategy, maturity, soccer, talent identification, youth athletes

## Abstract

**Aim:**

This study conducted the first scoping review with evidence gap map (EGM) on bio-banding in football, a field that has received increasing attention. Although previous studies have investigated technical and physical performance, including physiological outcomes, these findings have not yet been comprehensively reviewed. Hence, this review aimed to synthesize the existing evidence, identify research gaps, and inform future research priorities.

**Methods:**

This scoping review followed the PRISMA Extension for Scoping Reviews (PRISMA-ScR) guidelines. The databases searched were Web of Science, Scopus, SPORTDiscus, and ScienceDirect, and the searches were conducted on 29 January 2026. Eligible studies were original articles published in English or Spanish that investigated bio-banding in youth football players aged 11–15 years, with no restriction on publication year. Data on study characteristics, participant information, and study outcomes were extracted and synthesized to develop the evidence gap map (EGM).

**Results:**

A total of 47 records were identified, of which 20 met the selection criteria. Publications on bio-banding have increased markedly in recent years. Technical performance and psychological outcomes were the most frequently investigated themes, whereas no studies examined injury outcomes. The majority of studies involved male players and were conducted in Europe, highlighting the need for research involving female players, stakeholders, and more geographically diverse populations.

**Conclusion:**

Bio-banding is a promising strategy for talent identification and player development. Current evidence suggests that it may reduce maturity-related differences while supporting technical, tactical, and psychological development. However, important evidence gaps remain, particularly regarding physiological outcomes, female players, non-European populations, and diverse study designs. These findings provide practical guidance for coaches and practitioners and highlight priorities for future research.

## Introduction

Bio-banding is a method for grouping youth players within a specific chronological age (CA) range (11 to 15 years old) into “bands” or groups based on their estimated biological maturity status during specific competitions and/or training sessions (Malina et al., 2019). In the early 20th century, the concept of grouping children based on their biological maturity rather than CA was proposed. C. Ward Crampton (Crampton, 1908) proposed identifying physiological age by estimating the development of pubic hair (PH) in boys. Rotch (Rotch, 1908; Rotch, 1909) suggested grouping boys and girls in athletics by identifying their anatomical age based on the radiographic assessment of the carpal bones. Additionally, Baldwin (1916) assessed the physiological maturity of girls by estimating the development of menstrual flow, breast enlargement, and the appearance of subcutaneous fat and axillary hair. To date, bio-banding is used to group young players based on biological maturation thresholds (Cumming et al., 2017; Malina et al., 2019; Towlson and Cumming, 2022), which are related to the timing of the adolescent growth spurt (Towlson and Cumming, 2022).

In boys’ soccer, the application of bio-banding has attracted the greatest interest (Towlson and Cumming, 2022). Youth players are usually grouped based on their CA in competitions and training sessions; however, this grouping method overlooks the variation in individual biological maturity. For instance, some individuals mature earlier or later than their peers (Cumming et al., 2017). This grouping approach may lead coaches to select early-maturing players, as they possess more advanced physical characteristics (e.g., taller and stronger) and demonstrate better performance (e.g., greater speed and power) in competition. In addition, early-maturing boys may possess more adaptive motivational characteristics and higher levels of physical self-perception (Cumming et al., 2012, 2017). Hence, bio-banding is a key strategy proposed to address the influence of differences in physical development among youth players, reduce inequity in competition, and identify talent (Cumming et al., 2017). For instance, a 2022 survey conducted among leading European soccer academies revealed that 66% had integrated bio-banding into their routine training practices (Towlson and Cumming, 2022; Yaghoubinia et al., 2022). In addition, previous research has shown that players reported positive experiences following participation in bio-banded competitions and recommended that bio-banding be incorporated into the existing competition structure of the Premier League (Cumming et al., 2018). However, bio-banding has been less applied in women’s football (Towlson and Cumming, 2022). Previous research indicated that early-maturing girls typically outperform their late-maturing peers in absolute strength tests, whereas differences in performance among girls of different maturity statuses are negligible in tests of speed, agility, and power (Baxter-Jones et al., 2002; Cumming et al., 2017; Malina et al., 2004). Notably, early-maturing girls also present less adaptive psychological characteristics and lower self-esteem (Cumming et al., 2017; Petersen and Taylor, 1980).

It is worth mentioning that bio-banding serves as a supplement rather than a replacement for CA-based groups (Arroyo-Moya, 2023; Malina et al., 2019), and there are two non-invasive and feasible anthropometric methods commonly applied for assessing maturity (Cumming et al., 2017). The first method determines maturity offset, estimating the number of years a player is from reaching peak height velocity (PHV)—and classifies youth players into three categories based on maturation timing: pre-PHV, post-PHV, and on-PHV. The second method is the Khamis–Roche method (Khamis and Roche, 1994), which uses the percentage of predicted adult height (PAH) attained to bio-band players, classifying them into different categories based on their maturing status (Cumming et al., 2017), for example, ≥85.0 and <90.0%, or ≥90.0 and <95.0% (Malina et al., 2019). Although the two methods are widely used, one study found that the majority of football practitioners surveyed had adopted bio-banding and generally preferred the Khamis–Roche method (Khamis and Roche, 1994) for player grouping, perceiving it as the most valuable approach for maturity-matched grouping and particularly beneficial for late-maturing players or those who had passed their PHV (Towlson et al., 2023).

To date, bio-banding has been increasingly applied. However, research on bio-banding in football is relatively scattered, highlighting the need for a comprehensive review to integrate and analyze the various effects (i.e., physical, technical, tactical, physiological, psychological, and anthropometric parameters) of bio-banding on youth football players. Hence, this study aimed to conduct a scoping review of the existing literature on bio-banding in football and provide football practitioners and coaches with a comprehensive understanding of bio-banding in football and different perspectives on talent identification. In addition, according to our investigation, this study is the first scoping review with EGM of bio-banding in football. This review aimed to comprehensively search the peer-reviewed literature related to bio-banding in football, identify the research gaps, and guide future research on the topic.

## Methods

### Protocol and registration

This scoping review followed the PRISMA extension for Scoping Reviews (PRISMA-ScR), proposed by Tricco et al. (2018), and considered the Joanna Briggs Institute (JBI) methodology (Peters et al., 2020).

### Search strategy

This search aimed to comprehensively identify all literature related to bio-banding in football, with no restrictions on study design. Searches were conducted on 29 January 2026, and the following databases were used: Web of Science, Scopus, SPORTDiscus, and ScienceDirect. Additionally, the research strategy used Boolean operators to optimize the search, and the following keywords were used: bio-banding AND football OR soccer.

### Selection process

The process involved manual removal of duplicate articles and screening the retrieved articles in two stages: (i) screening titles and abstracts and (ii) analyzing the full texts of those that passed the first screening stage to determine whether to include or exclude them.

The first author and the corresponding author analyzed the articles based on the inclusion and exclusion criteria (Table 1), and the articles that were included or excluded were all reviewed by the co-authors. In the event of disagreements between co-authors, articles were re-analyzed until a consensus was achieved. It is worth noting that only peer-reviewed articles published in English or Spanish were included because these articles were indexed in core databases and satisfied the inclusion criteria of this review. When retrieved articles from the four databases lacked an abstract, reviewers performed additional searches via the East China Normal University Library and Loughborough University Library. If full texts remained unavailable after these searches, the articles were excluded; otherwise, the full texts were analyzed and eligibility was assessed.

**Table 1:** Inclusion and exclusion criteria for selecting studies on bio-banding in football.

Inclusion criteria	Exclusion criteria
Bio-banding in football (soccer) Original peer-reviewed journals 11 to 15 youth football players Relevant themes about bio-banding in football (e.g., psychology, physiology, technical performance, tactical performance, physical performance, and anthropometry) Written in English or Spanish All original research types All years of publication	Results combined football with other sports

### Critical appraisal of the included studies

A critical appraisal of all included studies was conducted using three study design-specific appraisal tools for quantitative, qualitative, and mixed-methods studies. The appraisal was undertaken to assess the methodological quality of the included studies and to provide additional context for interpreting the available evidence. The results of the critical appraisal are presented in the Supplementary Files. The appraisal findings were not used to exclude studies but were considered when interpreting the evidence and identifying methodological limitations and priorities for future research.

### Data extraction process

The primary author developed the data extraction form, which was reviewed by co-authors. Any disagreements were resolved by joint re-analysis until a consensus was achieved. All relevant information was organized and coded using a proprietary Microsoft Excel worksheet, and the extracted items included the author, journal, title, abstract, publication year, and geographical location. All information extracted from the included studies was classified into relevant domains systematically, and the outcomes were categorized as follows: number of participants, age (11 to 15 years old), sex (male/female), and sources of participants. In addition, the primary author determined and categorized the research themes by analyzing the titles and abstracts and developed a categorization of themes from the included studies (Table 2).

**Table 2:** Research themes and descriptors.

Theme	Descriptor
Psychology	Research that analyzed the psychological impact of bio-banded matches on players
Physiology	Research that analyzed the physiological impact of bio-banded matches on players
Technical performance	Research that investigated the technical skills of youth football players in bio-banded matches
Tactical performance	Research that investigated the tactics of youth football players in bio-banded matches
Physical performance	Research that investigated the physical performance of youth football players in bio-banded matches
Anthropometry	Research that investigated the methods for performing bio-banding

Due to the limited number of studies and participant sources, participants were mainly recruited from clubs or academies, and their performance levels were influenced by differences in biological maturity to a certain degree. Therefore, it was difficult to classify the performance levels of participants. In addition, as this review aimed to identify talent more accurately for coaches, classifying performance levels was inconsistent with the purpose of the review. Therefore, the performance levels of participants were not classified in this study.

### Data charting and synthesis methods

All data extracted were organized into a single Microsoft Excel worksheet and presented in different charting forms. For example, line graphs were used to describe continuous data (i.e., the year of publication), and pie charts were used to describe categorical data (i.e., populations and themes). In addition, to present an overview of the current body of knowledge on bio-banding and to identify research gaps for future research, an evidence gap map was developed. The EGM is a product of systematic evidence synthesis, which is used to present the existing evidence associated with specific research questions (see Table 3).

**Table 3:** Summary table of all included studies.

Study	Author(s)	Publication year	Country	Theme	Population	Study design	Main findings
Towlson et al. (2021a)	Christopher Towlson, Grant Abt, Steve Barrett, Sean P. Cumming, Frances Hunter, Ally Hamilton, Alex Lowthorpe, Bruno Goncalves, Martin Corsie, and Paul Swinton	2021	UK	Technical performance	Professional players	Quantitative	Early players displayed more effective collective behaviors, with bio-banding enhancing their integration into team dynamics when mixed with late players while having limited impact on late players; additionally, smaller pitch areas increased players’ tactical behaviors and technical performance.
Lüdin et al. (2022)	Dennis Lüdin, Lars Donath, Stephen Cobley, and Michael Romann	2022	Switzerland, Germany, and Australia	Technical performance, tactical performance, and physiology	Professional players	Quantitative	Compared to CA competition, in bio-banded competition, late-maturing players are provided with more opportunities to show technical-tactical skills, and early-maturing players face a higher physiological challenge in bio-banded competition.
Towlson et al. (2022)	Christopher Towlson, Calum MacMaster, Bruno Gonçalves， Jaime Sampaio, John Toner, Niall MacFarlane, Steve Barrett, Ally Hamilton, Rory Jack, Frances Hunter, Amy Stringer, Tony Myers, and Grant Abt	2022	UK and Portugal	Technical performance and tactical performance	Professional players	Quantitative	Bio-banding has a limited effect on the technical and tactical performance of young academy players in small-sided games, and small-sided games reduce maturity-related differences.
Abbott et al. (2019)	Will Abbott, Stuart Williams, Gary Brickley, and Nicholas J Smeeton	2019	UK	Technical performance and physical performance	Professional players	Quantitative	Bio-banded competition places a unique technical demand upon athletes of differing maturation groups when compared to chronological formats, without reducing the physical demands.
Salter et al. (2025)	Jamie Salter, Dale Forsdyke, Lukas Arenas， Zoe Dawson， Michael King, Naomi Myhill, James Robinson, Christopher McLaren-Towlson，Matt Springham， Laura Walsh, Sarah Mallinson-Howard， Steve Barrett	2025	UK and Germany	Technical performance and physical performance	Professional players	Quantitative	Bio-banding was associated with altered physical and technical demands and may provide an appropriate competition format, exposing players to different developmental challenges, which may support more equitable and balanced experiences.
Towlson and Cumming (2022)	Christopher Towlson and Sean P. Cumming	2022	UK	Technical performance, tactical performance, physical performance, psychology, and anthropometry	Professional players	Qualitative	Bio-banding can alleviate differences in athletes’ physical morphology within groups, reduce technical and tactical performance disparities caused by maturity differences in competition, and develop the psychological abilities of late-maturing players.
Cumming et al. (2018)	Sean P. Cumming, Daniel J. Brown, Siobhan Mitchell, James Bunce, Dan Hunt, Chris Hedges, Gregory Crane, Aleks Gross, Sam Scott, Ed Franklin, Dave Breakspear, Luke Dennison, Paul White, Andrew Cain, Joey C. Eisenmann, and Robert M. Malina	2018	UK and US	Technical performance, tactical performance, physical performance, and psychology	Professional players	Qualitative	All players reported positive experiences of participating in the bio-banded tournament. Early-maturing players perceived greater physical contact in bio-banded matches, which compelled them to adapt their playing styles and place greater emphasis on technical and tactical execution. Conversely, late-maturing players found the level of physical confrontation to be lower, and they highly valued the opportunities such competitions offered to practice and develop their technical, physical, and psychological capabilities.
Towlson et al. (2023)	Christopher Towlson, Demi Jo Watson, Sean P. Cumming, Jamie Salter, and John Toner	2023	UK	Technical performance, tactical performance, physical performance, psychology, and anthropometry	Coaches	Qualitative and quantitative	The majority of practitioners use bio-banding (preferring the Khamis–Roche method) as it improves player assessment, benefits late-maturing athletes, and aids development while facing barriers like coach buy-in and logistical issues.
Arroyo-Moya (2023)	Arroyo Moya Wilson	2023	US	Technical performance, tactical performance, physical performance, and psychology	Professional players	Qualitative	Bio-banding enables athletes at different maturation stages to exhibit a wide range of physical and technical-tactical performances, with positive benefits for all maturation groups. Meanwhile, bio-banding is clearly an effective tool for supporting athletes’ psychological development. However, the mechanisms through which bio-banding operates in talent identification remain to be further explored.
King et al. (2025)	Michael King, Steve Barrett, Ross Mclellan, John Cox, Matthew Brown, Sam Mackenzie, and Chirstopher Towlson	2025	UK	Technical performance, physical performance, and psychology	Professional players	Quantitative	Smaller team sizes and maturity-matched bio-banding formats enhanced technical actions and psychological characteristics, particularly among early-maturing players.
Reeves et al. (2018)	Matthew J. Reeves, Kevin J. Enright, Jack Dowling, and Simon J. Roberts	2018	UK	Technical performance, tactical performance, and psychology	Professional players, coaches, and parents	Qualitative	Bio-banding is generally beneficial overall. It protects late-maturing players, challenges early-maturing players, and reduces unfairness caused by maturity differences. However, limited understanding, poor communication, timing issues, and logistical difficulties are the biggest barriers to implementation.
Romann et al. (2020)	Michael Romann, Dennis Lüdin, and Dennis-Peter Born	2020	Switzerland	Technical performance, tactical performance, physical performance, and anthropometry	Professional players	Quantitative	Bio-banding reduced the distances covered in jogging, running, and high-speed running in youth players and anthropometric variables while shortening the mean time of ball possession per action and lowering pass accuracy—resulting in a faster-paced, more technically and tactically demanding game with reduced physical load.
Bradley et al. (2019)	Ben Bradley, David Johnson, Megan Hill, Darragh McGee, Adam Kana-ah, Callum Sharpin, Peter Sharp, Adam Kelly, Sean P. Cumming and Robert M. Malina	2019	UK and US	Technical performance, tactical performance, physical performance, and psychology	Professional players	Qualitative and quantitative	Players generally perceived bio-banding positively. Early maturers experienced greater physical or technical challenge, and late maturers reported greater opportunities for leadership and skill expression.
Towlson et al. (2021b)	Christopher Towlson, Calum MacMaster, Bruno Gonçalves, Jaime Sampaio, John Toner, Niall MacFarlane, Steve Barrett, Ally Hamilton, Rory Jack, Frances Hunter, Tony Myers, and Grant Abt	2021	UK and Portugal	Physical performance and psychology	Professional players	Quantitative	Maturity-matched bio-banding had limited effects on physical indicators, whereas maturity-mismatched grouping highlighted the psychological advantages of early-maturing players. Both bio-banding approaches demonstrated distinct strengths, with the Khamis–Roche method being more suitable for physical indicators and the Fransen method showing greater relevance for psychological indicators.
Robinson et al. (2025)	James Robinson, Dale Forsdyke, Lukas Arenas, Zoe Dawson, Michael King, Naomi Myhill, Christopher Towlson, Matt Springham, Laura Walsh, Sarah Mallinson-Howard, Steve Barrett, Jamie Salter	2025	UK and Germany	psychology	Professional players	Quantitative	In 11v11 soccer, bio-banding significantly improved talent experts’ quantitative ratings of competitiveness, confidence, decision-making, composure, and X-factor overall, especially benefiting less mature and on-time maturing players while slightly reducing early-maturing players’ perceived resilience and competitiveness.
Arede et al. (2024)	Jorge Arede, John M. Radnor, Oliver Gonzalo-Skok, and Nuno Leite	2024	Portugal, UK, and Spain	Physical performance	Professional players	Quantitative	During biological age-based competition, players covered less distance, performed fewer decelerations, achieved lower average speeds, had fewer body impacts, and recorded lower heart rates (HR) peaks compared to chronological age-based competition. Furthermore, no significant differences were found between the two formats in terms of spatial exploration index.
MacMaster et al. (2021)	Calum MacMaster, Matt Portas, Guy Parkin, Sean P. Cumming, Chris Wilcox, and Christopher Towlson	2021	UK	Physical performance and anthropometry	Professional players	Quantitative	Compared with chronological age grouping, bio-banding significantly reduces variations in anthropometric measures and partially lessens variations in physical fitness yet exerts limited impact on variations in functional movement.
Segueida-Lorca et al. (2022)	Álvaro Segueida-Lorca1, Joel Barrera, Luis Valenzuela-Contreras, and Tomás Herrera-Valenzuela	2022	Chile and Portugal	Anthropometry	Professional players	Quantitative	In young male footballers classified by bio-banding, maturity level significantly affects body composition, with the greatest increases in height and body mass, followed by muscle and bone mass gains, smaller rises in adipose mass and skeletal muscle mass index, and minimal changes in summed skinfolds.
Salter et al. (2023)	Jamie Salter, James Black, James Mallett, Steve Barrett, Christopher Towlson, Jonathan D. Hughes, and Mark De St Croix	2023	UK	Physiology	Professional players	Quantitative	Bio-banding reduced neuromuscular fatigue and perceived intensity compared to chronological-age grouping, especially mitigating large increases in leg stiffness and stride-length reductions in pre- and circa-PHV academy soccer players.
Barrett et al. (2022)	Steve Barrett, Kieran Short, Alex Lowthorpe, Paul Swinton, Patrick Maughan, Ally Hamilton, Frances Hunter, Christopher Towlson	2022	UK	Physiology	Professional players	Quantitative	Under bio-banded conditions, pre-PHV players reported higher subjective exertion ratings than post-PHV players in small-sided games; in addition, the overall perceived exertion was generally higher when players of different maturational statuses were evenly mixed.

## Results and discussion

### Study identification and selection

An initial search yielded a total of 47 records. After removing 12 duplicates, the remaining 35 records were screened for relevance based on titles and abstracts, and 11 ineligible records were excluded because these studies did not investigate youth football players. Five records were removed for other reasons. The remaining 19 records met the inclusion criteria and passed the eligibility assessment. Additionally, we screened the reference lists of the included articles and identified one article that was considered relevant to this review. Ultimately, 20 studies were included in the review, and the PRISMA flowchart of the study identification and selection process is presented in Figure 1.

**Figure 1:**
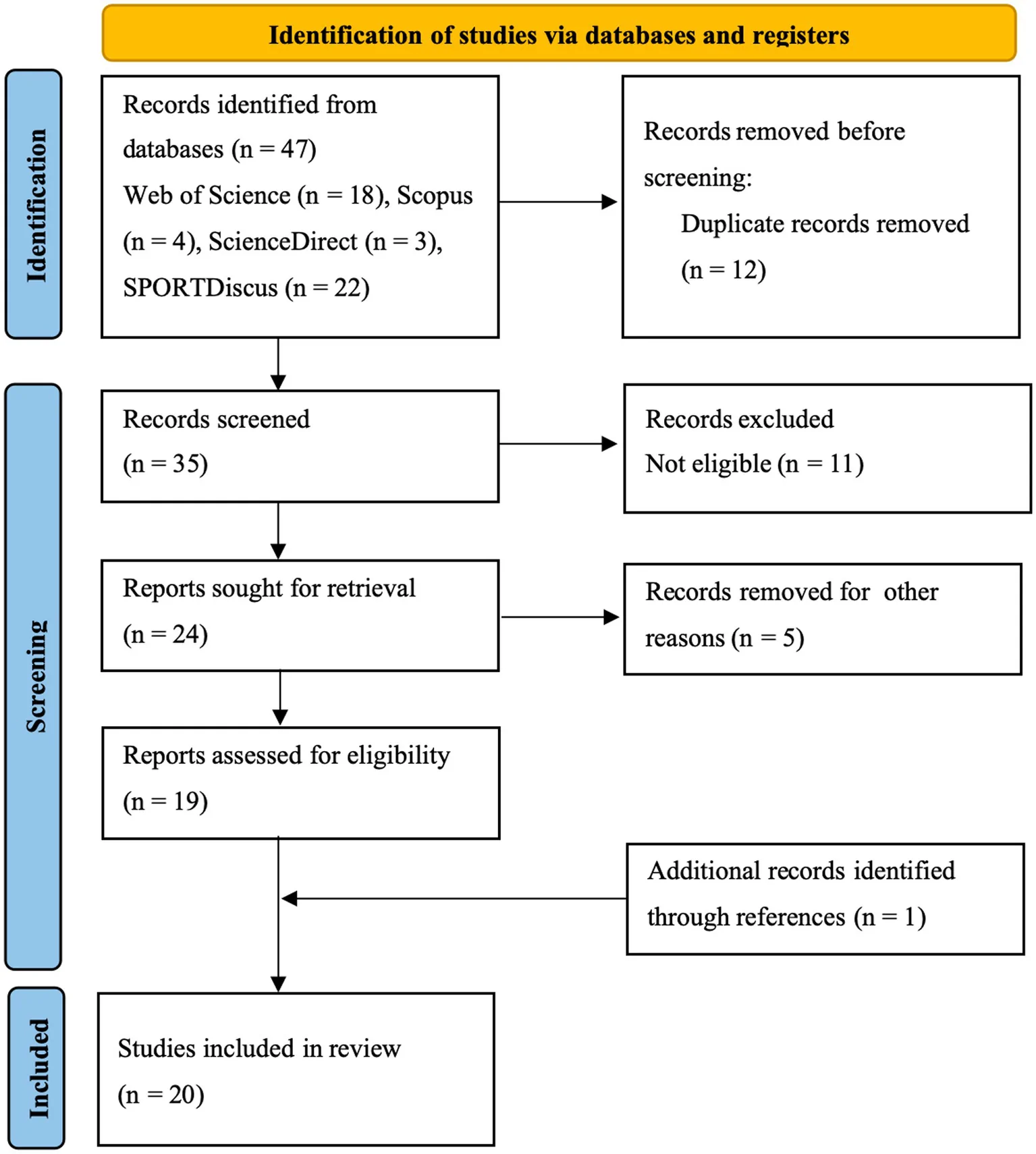
PRISMA 2020 flow diagram.

### Publication tendencies

Although the concept of grouping children based on their biological maturity has been proposed for many years (Crampton, 1908), the number of studies focusing on bio-banding in football has increased significantly in recent years. Since 2018, the number of studies related to bio-banding in football has shown an upward trend and peaked in 2021 (*n* = 4) and 2022 (*n* = 4); however, studies related to this subject are still in single digits (Figure 2). There are valid reasons to explain the increasing number of studies on bio-banding in football in recent years. In terms of competition practice, the English Premier League has long been at the forefront of applying growth and maturation research to long-term athlete development (Cumming et al., 2017). Meanwhile, coaches and football practitioners have a long-standing need to identify and select talented players, and bio-banding can help them better identify talent to a certain extent. However, the number of studies focusing on bio-banding in football (*n* = 20) is insufficient. This indicates that many potential effects of bio-banding may remain unexplored, and further research is needed to promote the development of youth football in the future.

**Figure 2:**
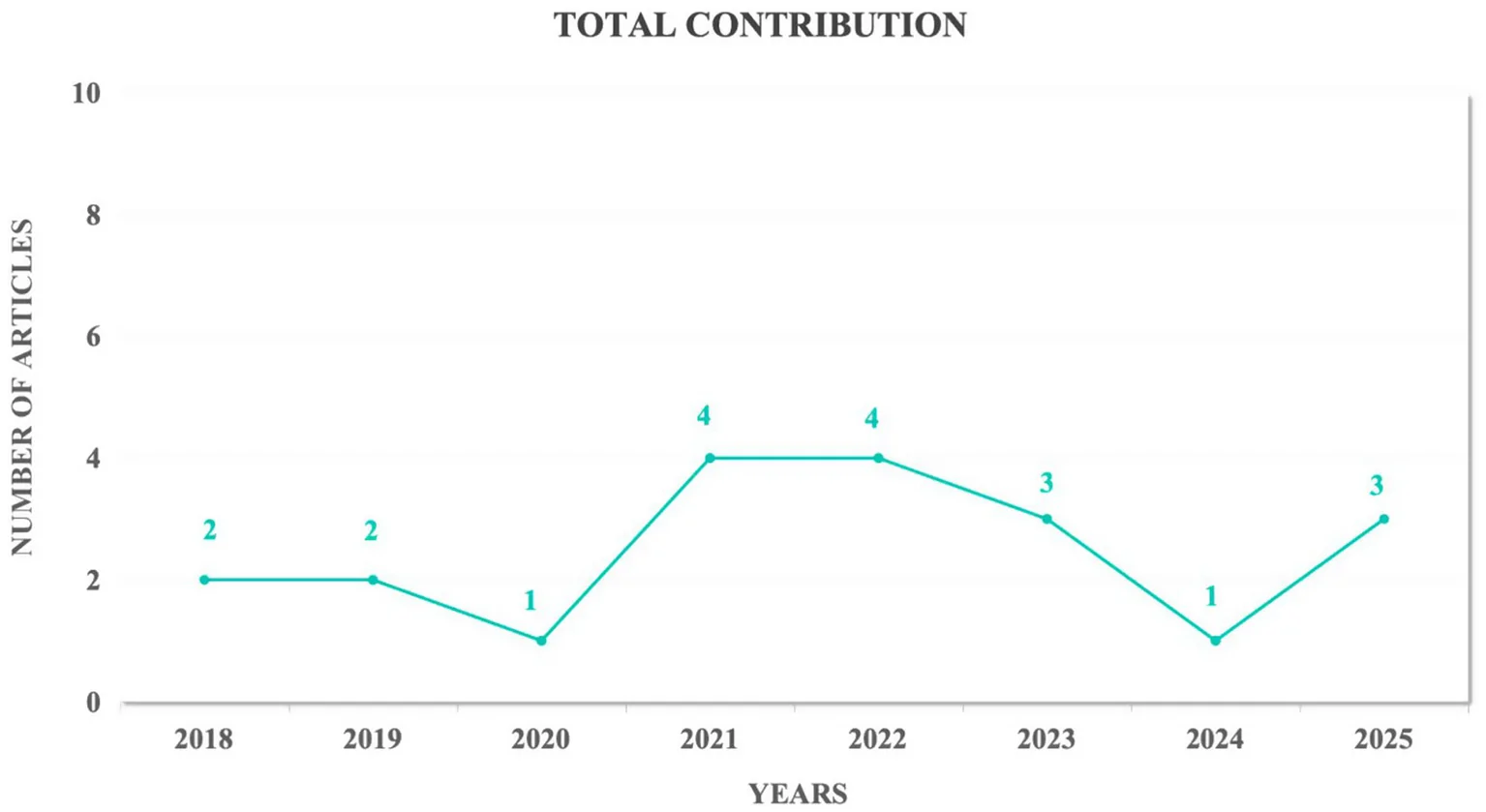
Number of studies on bio-banding in football published over the years.

### Study characteristics

The majority of research studies focused on 11- to 15-year-old players, while only one study included participants aged U12 to U17 and U19; hence, this review adopted data from football players aged 11–15 years. For studies that included participants outside the 11–15 age range, we extracted only data explicitly reported for the 11–15 age subgroups. Data presented at aggregated age group levels were not extracted. It is worth explaining that the age range of 11–15 years was selected as it corresponds to the typical window of the adolescent growth spurt and PHV, when maturity-related differences are most pronounced. Notably, only one study focused on female participants. Therefore, research on bio-banding in football that focuses on female participants is insufficient. In addition, one study used stakeholders (i.e., professional football academy staff, youth players, and parents/carers) as participants, and another study focused on practitioners (Figure 3).

**Figure 3:**
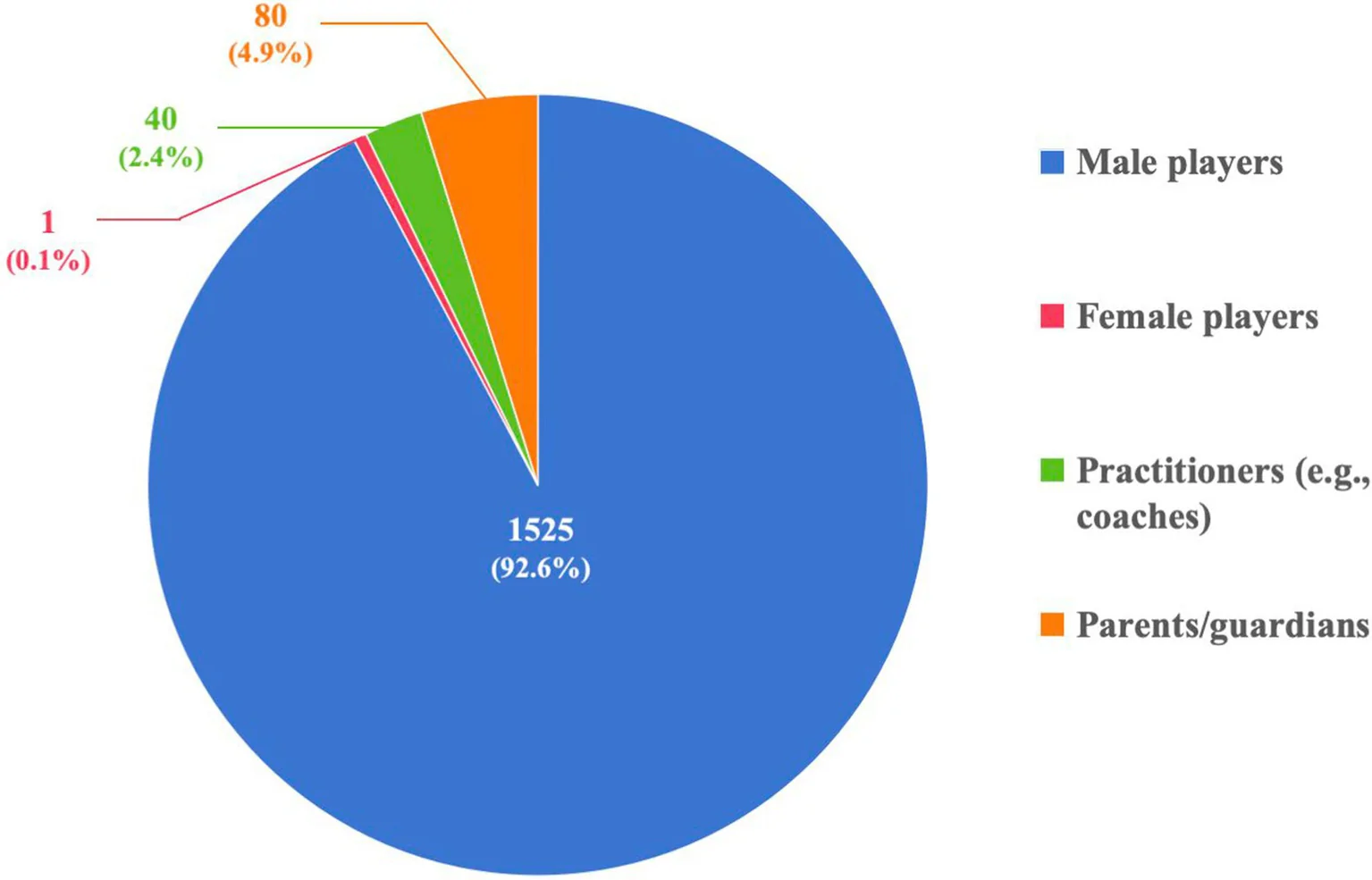
Participant group distribution.

### The theme of publications

The included studies cover 6 different themes (Figure 4). Among studies using youth players as participants, four studies investigated the effect of bio-banding on a single element (i.e., technical, psychological, or body composition), and 12 studies investigated its effects based on multiple elements; for example, a study investigated the effect on physical and psychological indicators (Towlson et al., 2021b). Additionally, three studies explored the subjective perceptions of bio-banding among players, practitioners, and stakeholders.

**Figure 4:**
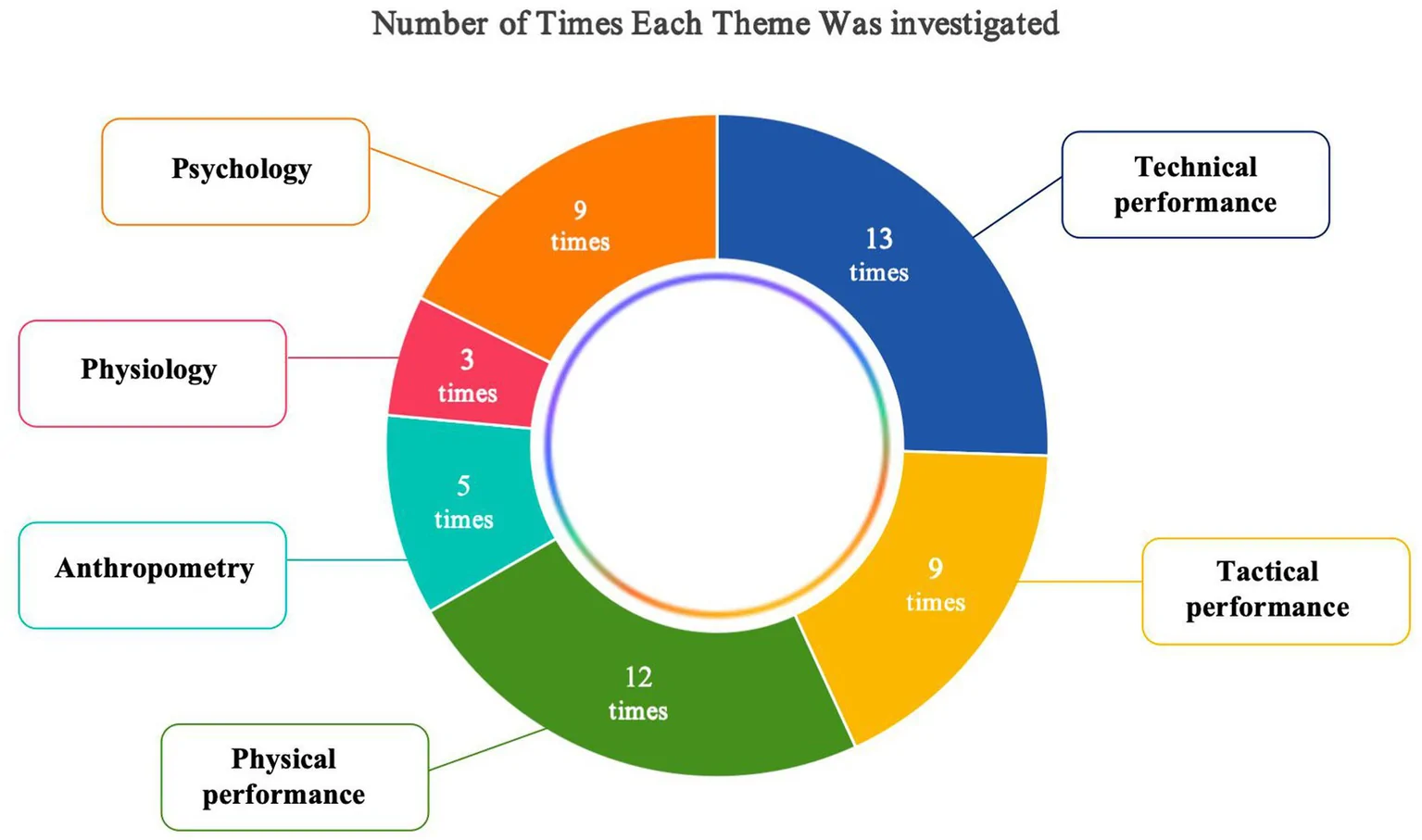
Number of times each theme was investigated.

Of these studies, technical performance was the first major research theme (*n* = 13). Physical performance followed, with 12 studies. Studies on psychology and tactical performance were ranked third with nine publications. Although research on bio-banding in football is still in development, the distribution of research themes has become diverse (Figure 4).

### Technical performance

Thirteen studies analyzed technical variables (e.g., passing, shooting, and dribbling). Of these studies, 11 were conducted with youth football players, and one recruited stakeholders (i.e., players, staff, and parents/guardians) as participants. In addition, one study investigated practitioners’ perceptions (e.g., coaches, sport scientists, and injury specialists).

Seven quantitative studies conducted matches and reported pitch dimensions. Of these studies, two were conducted on full-sized standard grass pitches (100 × 64 m) (Abbott et al., 2019; Salter et al., 2025), while the other studies used small-sided games (King et al., 2025; Lüdin et al., 2022; Romann et al., 2020; Towlson et al., 2021a). On full-sized standard grass pitches (100 × 64 m), players exhibited a longer average ball-possession duration (Salter et al., 2025), and early-maturing players performed more short passes and fewer dribbles, whereas late-maturing players performed significantly more tackles and fewer long passes during bio-banded competition (Abbott et al., 2019). However, in small-sided games, bio-banding increased the number of set pieces and reduced the average time of ball possession (Romann et al., 2020). It is worth noting that Towlson et al. (2021a) investigated passing networks in different pitch dimensions based on bio-banding and observed that as relative pitch size increased, the effect of players’ maturity differences on performance became more significant, and early-maturing players showed more effective collective behaviors than late-maturing players. Additionally, King et al. (2025) analyzed the effect of team size on youth football players within bio-banded formats and found that smaller team size and maturity-matched bio-banding formats could enhance technical actions.

Overall, in bio-banded match conditions, full-sized pitches and small-sided pitches produce distinct differences in players’ technical performance. For instance, in small-sided games, the average time of ball possession was reduced. One possible explanation is that the effects of bio-banding might be moderated by the characteristics of the playing environment. Full-sized pitches provide greater spatial availability and may allow players more time and space for possession, whereas small-sided games typically involve greater density, potentially increasing spatial pressure and reducing opportunities for prolonged possession. Hence, the effects of bio-banding on ball possession may depend not only on players’ maturity but also on pitch dimensions and player density. However, these explanations remain hypotheses and require further investigation.

In addition to objective assessment of players’ technical performance, their perceptions provide valuable insight into the implementation and evaluation of bio-banding. For instance, players reported that participation in bio-banded competition contributed to significant improvements in their technical performance (Reeves et al., 2018; Bradley et al., 2019). Notably, Towlson et al. (2023) investigated soccer academy practitioners’ perceptions of bio-banding and found that practitioners believed that bio-banding could improve the accuracy of technical talent assessment and considered the promotion of technical development to be one of the primary objectives of its implementation.

Technical performance as a theme received the most attention, which indicates that it is a key factor for talent identification. In addition, practitioners might better understand the technical characteristics of players in bio-banded matches and provide approaches to help coaches optimize training plans by investigating youth football players’ technical performance under bio-banding. For example, coaches could change the pitch dimensions or team size (e.g., 4v4 and 5v5 formats) to enhance technical performance among players at different maturity levels.

### Tactical performance

A total of nine studies investigated tactical performance, targeting male youth football players. Overall, bio-banded formats have a limited effect on tactics. For instance, Towlson et al. (2022) found that in maturity-matched games, bio-banding had no significant effect on tactical outputs for circa-PHV and pre-PHV players, and Romann et al. (2020) reported that compared to CA matches, bio-banded matches increased the number of duels; however, there were no significant differences in the remaining tactical parameters between bio-banded and CA conditions. Notably, some studies demonstrated that bio-banding exerted greater influence on post-PHV players (Arroyo-Moya, 2023; Towlson et al., 2021a; Towlson et al., 2022). Nevertheless, players perceived that bio-banding facilitated changes in tactical behavior (Arroyo-Moya, 2023; Cumming et al., 2018; Bradley et al., 2019).

Generally, the conclusions of the research regarding tactical performance were not as comprehensive as those regarding technical performance. For instance, defensive actions, offensive actions, and decision-making might be potential areas for further investigation.

### Physical performance

A total of 12 studies focussed on this theme. One study analyzed players’ within-group variance under bio-banding using physical fitness measures and functional movements and postural assessments (MacMaster et al., 2021). The results showed that bio-banding reduced the within-group variance of players’ physical fitness characteristics (MacMaster et al., 2021; Bradley et al., 2019). Four studies investigated physical performance by comparing bio-banded matches with CA matches (Abbott et al., 2019; Arede et al., 2024; Romann et al., 2020; Salter et al., 2025), and two studies compared maturity-matched games (e.g., pre-PHV vs. pre-PHV) with maturity-mismatched matches (e.g., post-PHV vs. pre-PHV) (King et al., 2025; Towlson et al., 2021b). These studies analyzed the physical indicators among players and found that there were significant differences in physical performance between youth players categorized into different maturity groups (Abbott et al., 2019; Salter et al., 2025). Both early- and late-maturing players perceived that bio-banding could create a more physically equitable competitive environment (Cumming et al., 2018; Bradley et al., 2019). In addition, Arede et al. (2024) analyzed the effects of bio-banding on spatial exploration behavior in 7v7 match play, and the results showed that the spatial exploration index did not differ significantly between the two competition formats (Arede et al., 2024).

Due to variations in samples and study design, the results of these studies do not reveal many commonalities. Hence, more research studies on this theme are needed in the future to reveal more commonalities in physical performance and enable practitioners to better understand bio-banding.

### Psychology

A total of nine studies investigated this area. Six studies were conducted with youth football players, and one study recruited stakeholders (i.e., professional football academy staff, youth players, and parents/carers) as participants. Bradley et al. (2019) reported that players generally held positive attitudes toward bio-banding. The player-specific studies evaluated key psychological attributes: ‘Confidence’, ‘Competitiveness’, ‘X-Factor’, and ‘Positive Attitude’ (King et al., 2025; Towlson et al., 2021b), and one study used the Hull Soccer Behavioral Scoring Tool (HSBST) to analyze youth players’ psychological attributes: ‘Resilience’, ‘Competitiveness’, ‘Confidence’, ‘Decision Making’, ‘Maintaining’, ‘Communication’, and ‘X-Factor’. The majority of psychological indicators in these studies were evaluated by professional academy scouts or coaches (King et al., 2025; Robinson et al., 2025; Towlson et al., 2021b), and the results showed that significant differences were observed in psychological indicators among players of different maturity statuses under bio-banding (Robinson et al., 2025; Towlson et al., 2021b) and that bio-banding could enhance players’ psychological development (Robinson et al., 2025). Notably, King et al. (2025) investigated the effect of team size on players’ psychological outcomes, and the results showed that with smaller team sizes (i.e., 4v4), more mature players exhibited higher psychological scores. In addition, Reeves et al. (2018) analyzed stakeholders’ perceptions of bio-banding and found that psychological factors were cited most commonly. Both early- and late-maturing players reported that their confidence was enhanced in bio-banding competitions (Cumming et al., 2018). It is worth noting that, based on interviews with practitioners, Towlson et al. (2023) found that practitioners believed that bio-banding could offer several psychosocial benefits for players.

Generally, research in this area was relatively comprehensive and contributed to both theory and practice, deepening football practitioners’ understanding of the psychological characteristics of youth players of different maturity statuses, identifying their respective psychological strengths, and thus enabling coaches to design more targeted training programs.

### Physiology

Three studies focused on this area among youth football players. Salter et al. (2023) and Barrett et al. (2022) examined the differential ratings of perceived exertion (dRPE) among players. Additionally, Salter et al. (2023) explored the acute impact of maturation on neuromuscular performance by comparing both chronological and bio-banded training formats. Although both studies found differences in perceived exertion between players of different maturity statuses, Lüdin et al. (2022) did not observe a simultaneously significant increase in other measured quantitative physiological indicators that could have mediated the higher RPE (i.e., total distance covered, high-speed running distance covered, and explosive distance covered). Hence, the reasons for the increased perceived exertion remain to be investigated.

The results of these studies involved dRPE or RPE among players and revealed that bio-banded training may enable practitioners to individualize training loads based on players’ maturity status, thereby reducing the potential physical strain associated with repeated high-intensity exercise (Salter et al., 2023). However, research in this area remains limited, and the potential mechanisms of the relationship between various indicators and athletic performance have not been completely analyzed.

### Anthropometry

In this area, five studies explored anthropometry among youth football players. One study focused on comparing body composition (e.g., muscle mass, bone mass, height, and body mass) in youth football players categorized by bio-banding (Segueida-Lorca et al., 2022). The majority of studies demonstrated that bio-banding reduced the within-group variance for anthropometric variables (MacMaster et al., 2021; Romann et al., 2020; Towlson and Cumming, 2022). It is worth noting that Towlson et al. (2023) reported that 80% of the practitioners surveyed had implemented bio-banding. Practitioners generally preferred the Khamis–Roche method (Khamis and Roche, 1994) for player grouping, perceiving it as the most valuable approach for maturity-matched grouping and particularly beneficial for late-maturing players or those who had passed their PHV.

Although research on this topic is relatively limited, the findings are largely consistent across studies, suggesting that these conclusions may be broadly applicable. However, further research on this theme in the future may help coaches to identify, select, and develop talented players.

### EGM of the included research

This scoping review developed an EGM (Figure 5) to synthesize the relevant available evidence. The EGM presents bio-banding research in football across research themes, participant groups, geographic regions, and study designs. Overall, the available evidence is concentrated on technical performance, tactical performance, physical performance, and psychology among male players, with the majority of studies conducted in Europe employing a quantitative research design. In contrast, research targeting female players, coaches/scouts, and parents/carers remains relatively limited. Geographically, research from Asia, Africa, and Oceania remains scarce compared with that from Europe and the Americas. Regarding research themes, studies on physiology remain relatively scarce, whereas technical, tactical, and physical performance have received the greatest research attention. In addition, qualitative and mixed-methods studies are underrepresented, indicating that research methodologies in this field remain relatively homogeneous. Taken together, these findings suggest that substantial evidence gaps persist in football bio-banding research, particularly with respect to specific participant populations, non-European regions, physiological outcomes, and the application of more diverse research designs. These areas warrant greater attention in future research.

**Figure 5:**
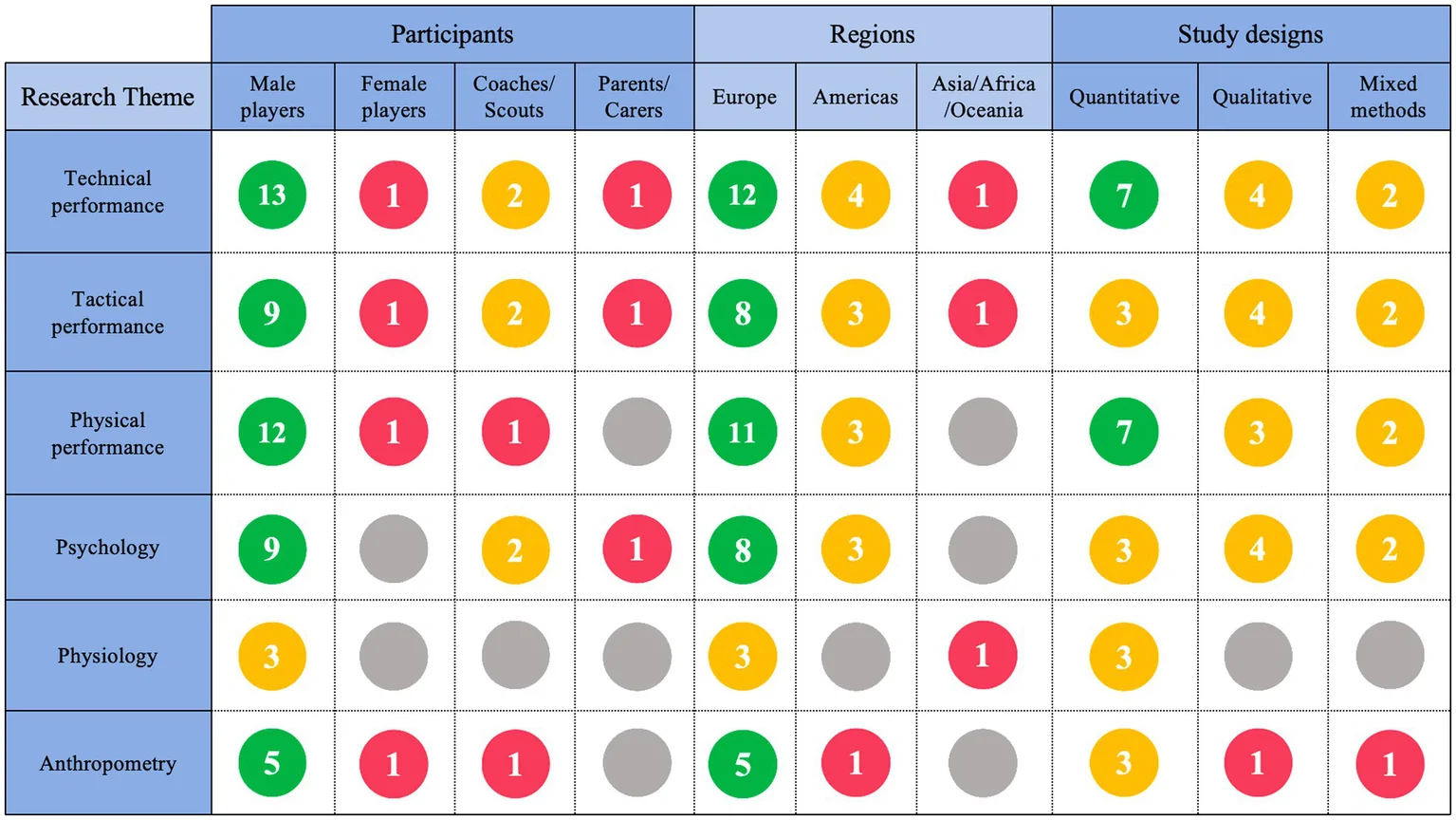
Evidence gap map of bio-banding in football. Each cell represents the number of included studies investigating the corresponding research theme within the respective participant group, geographical region, or study design. Green indicates themes investigated more than five times; yellow indicates themes investigated two to four times; red indicates themes investigated once only; and gray indicates themes that have not yet been investigated.

### What are the research gaps that need to be investigated in further research?

One of the main contributions of this research is to identify the research gaps in relation to the investigated populations and themes that need to be investigated in further studies. Additionally, this study presents the evidence gap map (Figure 5) to synthesize the evidence related to bio-banding in football and provide a visual presentation of the evidence. Regarding study regions, the majority of the included studies were conducted in Europe; hence, the generalizability of the current findings may be limited for populations outside Europe. Future research could extend beyond European settings and adopt cross-cultural and cross-geographic approaches to examine the effectiveness and benefits of bio-banding. In terms of research participants, limited research has targeted female populations, with only one study recruiting a single female participant as one of its subjects. This lack of investigation limits our understanding of the specific physiological, psychological, and performance characteristics of young female football players in the bio-banding environment. Additionally, the timing and characteristics of physical development differ between male and female football players; therefore, these differences might contribute to different performances between male and female football players in the bio-banding environment. Meanwhile, the same grouping methods (e.g., the Khamis–Roche method) may not have equivalent validity or feasibility in female populations. It is worth noting that focusing on women’s football may also contribute to the promotion of gender equality (Bao et al., 2025). Bio-banding as a potential method to identify talent might help coaches and other stakeholders select talented young players. However, because the majority of studies focus on boys, talented young female players are less likely to be selected and recruited. Only if bio-banding in girls’ football is investigated as comprehensively as boys’ football will talented young girls be identified more effectively and the sport be expected to develop more equitably. Notably, this study highlights the need for research into perceptions among coaches, referees, managers, and scouts. Only one included study focused on stakeholders (i.e., players, staff, and parents/guardians; Reeves et al., 2018). This indicates that these groups have been overlooked to date. Although players, as the core competition participants, receive the majority of research attention when bio-banding is considered as a talent identification approach, the research focus on coaches, referees, managers, and scouts should be emphasized to the same extent as that on players. They are integral to the game’s ecosystem, and their perceptions and decisions can influence players’ performance, talent selection, and outcomes. Therefore, research on these populations is highly significant for the optimization of training protocols, the fair conduct of competitions, and more effective identification of talented players, eventually benefiting the entire sport.

Concerning the research themes explored, this study found that performance analysis and psychology are research hotspots, as these two themes received the most academic attention. This enables football practitioners to understand the effects of bio-banding on the technical, tactical, and physical performances of young players, thereby optimizing training strategies and improving the accuracy of talent identification. Furthermore, it helps them gain insights into players’ psychological behaviors and variations within bio-banding settings and facilitates the development of targeted psychological attributes among players with varying maturation levels. However, according to the authors’ knowledge, it is surprising that no research has discussed the theme of medicine to date. Exploring medical aspects (e.g., injury epidemiology and risk factors) is of utmost importance for injury prevention and enhancing the sports performance of youth football players. Although the existing literature has reported that football practitioners believe that bio-banding could reduce the risk of sports injuries (Towlson et al., 2023), quantitative research examining this potential benefit remains limited. Injury outcomes represent a critical gap, as injury risk is relevant to the safety and feasibility of implementing bio-banding in youth football. Bio-banding could reduce maturity-related physical disparities; however, it remains unknown whether reducing such physical disparities translates into a safer competitive environment. For late-maturing players, bio-banding may reduce physical demands compared with competing against more mature players. Conversely, early-maturing players might be exposed to an environment that requires higher physical demands. Hence, it is difficult to determine whether bio-banding merely modifies the distribution of physical demands or also has significant implications for player safety. This represents an important limitation in evaluating the overall effectiveness and practical implementation of bio-banding in youth football.

### Strengths and limitations

This scoping review followed the guidelines of PRISMA-ScR (Tricco et al., 2018) and scoped the academic literature from formal research sources (i.e., peer-reviewed journals). However, this study has several limitations. Although we did not apply formal quality scoring, it should be acknowledged that the included studies vary in their methodological rigor. These variations may affect the overall strength of the evidence, and, therefore, some of the research gaps identified in this review could be attributable in part to the limitations of the primary studies rather than to a genuine lack of investigation on those topics. Readers are advised to interpret the mapped evidence with this caveat in mind. Furthermore, the search strategy used only the core terms ‘bio-banding’ AND ‘football OR soccer,’ without including related terms such as ‘maturity matching,’ ‘biological maturation,’ ‘relative age effect,’ or ‘growth and maturation.’ This relatively narrow approach yielded 47 records, which may have limited the retrieval of relevant studies that used different terminologies to describe similar concepts. Therefore, only a limited number of eligible studies were included, which may limit the comprehensiveness of the synthesized evidence.

In addition, the majority of the included studies were conducted in Europe, with only one study conducted in Chile (Segueida-Lorca et al., 2022). This high geographic concentration may result in findings biased toward the European region and may not reflect the bio-banding practices in other regions. Notably, except for two articles written in Spanish, all the remaining literature included was published in English. Thus, this bias may be further intensified, neglecting unique research findings from other countries and regions. Additionally, although the Khamis–Roche method (Khamis and Roche, 1994) and the maturity offset method are most commonly adopted in the included articles, and practitioners reported that coaches preferred the Khamis–Roche method (Khamis and Roche, 1994), various maturity assessment approaches may inevitably result in inconsistent outcomes. Hence, it is vital to conduct replicated studies using different methods to assess biological maturity to derive consistent conclusions. Finally, although we focused on players aged 11–15 years, the inclusion of one study covering U17 and U19 participants may have introduced heterogeneity into the evidence synthesis. This should be considered when interpreting the findings.

## Conclusion

According to the 20 included studies, this scoping review categorized the literature into 6 themes and developed an EGM to guide future research on the effects of bio-banding in youth football. Since 2018, the number of studies related to bio-banding in football has shown an upward trend over the years. Research has extensively covered a range of themes, including technical performance, tactical performance, physical performance, psychology, physiology, and anthropometry. Notably, technical performance and physical performance are the two dominant research themes in this field. This is likely because they enable coaches and scouts to better identify young football talents. In addition, preliminary evidence suggests that bio-banding has some potential benefits for reducing performance and anthropometric differences associated with biological maturity (Abbott et al., 2019; Arede et al., 2024; MacMaster et al., 2021; Salter et al., 2025), creating a more equitable competitive environment, thereby supporting youth player development. These findings might provide practical implications for practitioners. For coaches, existing research studies focus on technical performance, tactical performance, physical performance, and psychology, which could provide scientific guidance for talent selection and optimizing training plans for young football players. Regarding players, the gap in medical research also emphasizes the need to strengthen support in this area in the future, which could help players prevent injuries. However, due to existing research gaps, it is premature to make broad claims about the effectiveness of bio-banding. Notably, bio-banding serves as a supplement rather than a replacement for CA-based groups (Arroyo-Moya, 2023; Malina et al., 2019). Existing studies have suggested that practitioners use bio-banding only as an adjunct rather than a complete replacement for CA-based group competition and that it should be integrated into a diverse game program (Towlson and Cumming, 2022).

In general, this study reveals the complexity and diversity of bio-banding in football practice and contributes to the advancement of scientific research on youth football. Through reviewing the existing literature, this study develops the EGM, identifies research gaps that require further investigation, and provides guidance for expanding research themes, populations, and geographical locations. These findings offer valuable implications for football clubs, youth academies, sporting associations, coaches, scouts, and all sports professionals committed to optimizing talent identification and player development strategies. Meanwhile, this review also lays a solid foundation for future multidisciplinary collaborative research and practical application in this field.
