# Supplementary material for: Bio-banding in football: a scoping review with evidence gap map
Source: Front Psychol. 2026 Sep 16;17:1960364. doi: 10.3389/fpsyg.2026.1960364 (PMC13625182; doi:10.3389/fpsyg.2026.1960364)
Supplement: Supplementary file 1 [file Data_Sheet_1.PDF]

Quantitative Study Quality Assessment (Towlson et al., 2021)

	Assessment Criteria	Yes	No	Unclear	N/A
Domain 1: External Validity (Study Purpose & Population)					
1	Was the research question or objective clearly stated?	√			
2	Was the study population clearly defined and adequately described (e.g., inclusion/exclusion criteria)?	√			
3	Were the participants representative of the pre-specified target population?	√			
Domain 2: Internal Validity – Risk of Bias (Study Design & Execution)					
4	Was the study design appropriate for answering the research question (e.g., controlled design for interventions; cohort/case-control for exposures)?	√			
5	Were key potential confounders adequately controlled for (either in the design or statistical analysis)?	√			
6	Were the outcome/exposure measurements clearly defined, valid, and reliable (e.g., validated scales or objective measures)?	√			
7	Were the outcome/exposure measurements applied consistently across all participants?	√			
8	Was the sample size sufficiently powered to detect a statistically significant difference or association in the primary outcome?	√			
Domain 3: Data Completeness & Analysis (Information Bias)					
9	Were the data for key outcomes/exposures sufficiently complete (e.g., was attrition reported, <20%, or were missing data appropriately handled)?	√			
10	Were the statistical analyses appropriate (e.g., key assumptions tested, appropriate regression models used)?	√			
11	Were effect estimates (e.g., OR, RR, mean difference) with measures of precision (e.g., 95% CI) reported?	√			

Quantitative Study Quality Assessment (Lüdin et al., 2022)

	Assessment Criteria	Yes	No	Unclear	N/A
Domain 1: External Validity (Study Purpose & Population)					
1	Was the research question or objective clearly stated?	√			
2	Was the study population clearly defined and adequately described (e.g., inclusion/exclusion criteria)?	√			
3	Were the participants representative of the pre-specified target population?	√			
Domain 2: Internal Validity – Risk of Bias (Study Design & Execution)					
4	Was the study design appropriate for answering the research question (e.g., controlled design for interventions; cohort/case-control for exposures)?	√			
5	Were key potential confounders adequately controlled for (either in the design or statistical analysis)?	√			
6	Were the outcome/exposure measurements clearly defined, valid, and reliable (e.g., validated scales or objective measures)?	√			
7	Were the outcome/exposure measurements applied consistently across all participants?	√			
8	Was the sample size sufficiently powered to detect a statistically significant difference or association in the primary outcome?	√			
Domain 3: Data Completeness & Analysis (Information Bias)					
9	Were the data for key outcomes/exposures sufficiently complete (e.g., was attrition reported, <20%, or were missing data appropriately handled)?	√			
10	Were the statistical analyses appropriate (e.g., key assumptions tested, appropriate regression models used)?	√			
11	Were effect estimates (e.g., OR, RR, mean difference) with measures of precision (e.g., 95% CI) reported?	√			

Quantitative Study Quality Assessment (Towlson et al., 2022)

	Assessment Criteria	Yes	No	Unclear	N/A
Domain 1: External Validity (Study Purpose & Population)					
1	Was the research question or objective clearly stated?	√			
2	Was the study population clearly defined and adequately described (e.g., inclusion/exclusion criteria)?	√			
3	Were the participants representative of the pre-specified target population?	√			
Domain 2: Internal Validity – Risk of Bias (Study Design & Execution)					
4	Was the study design appropriate for answering the research question (e.g., controlled design for interventions; cohort/case-control for exposures)?	√			
5	Were key potential confounders adequately controlled for (either in the design or statistical analysis)?	√			
6	Were the outcome/exposure measurements clearly defined, valid, and reliable (e.g., validated scales or objective measures)?	√			
7	Were the outcome/exposure measurements applied consistently across all participants?	√			
8	Was the sample size sufficiently powered to detect a statistically significant difference or association in the primary outcome?	√			
Domain 3: Data Completeness & Analysis (Information Bias)					
9	Were the data for key outcomes/exposures sufficiently complete (e.g., was attrition reported, <20%, or were missing data appropriately handled)?	√			
10	Were the statistical analyses appropriate (e.g., key assumptions tested, appropriate regression models used)?	√			
11	Were effect estimates (e.g., OR, RR, mean difference) with measures of precision (e.g., 95% CI) reported?	√			

Quantitative Study Quality Assessment (Abbott et al., 2019)

	Assessment Criteria	Yes	No	Unclear	N/A
Domain 1: External Validity (Study Purpose & Population)					
1	Was the research question or objective clearly stated?	√			
2	Was the study population clearly defined and adequately described (e.g., inclusion/exclusion criteria)?	√			
3	Were the participants representative of the pre-specified target population?	√			
Domain 2: Internal Validity – Risk of Bias (Study Design & Execution)					
4	Was the study design appropriate for answering the research question (e.g., controlled design for interventions; cohort/case-control for exposures)?	√			
5	Were key potential confounders adequately controlled for (either in the design or statistical analysis)?	√			
6	Were the outcome/exposure measurements clearly defined, valid, and reliable (e.g., validated scales or objective measures)?	√			
7	Were the outcome/exposure measurements applied consistently across all participants?	√			
8	Was the sample size sufficiently powered to detect a statistically significant difference or association in the primary outcome?		√		
Domain 3: Data Completeness & Analysis (Information Bias)					
9	Were the data for key outcomes/exposures sufficiently complete (e.g., was attrition reported, <20%, or were missing data appropriately handled)?	√			
10	Were the statistical analyses appropriate (e.g., key assumptions tested, appropriate regression models used)?	√			
11	Were effect estimates (e.g., OR, RR, mean difference) with measures of precision (e.g., 95% CI) reported?	√			

Quantitative Study Quality Assessment (Salter et al., 2025)

	Assessment Criteria	Yes	No	Unclear	N/A
Domain 1: External Validity (Study Purpose & Population)					
1	Was the research question or objective clearly stated?	√			
2	Was the study population clearly defined and adequately described (e.g., inclusion/exclusion criteria)?	√			
3	Were the participants representative of the pre-specified target population?	√			
Domain 2: Internal Validity – Risk of Bias (Study Design & Execution)					
4	Was the study design appropriate for answering the research question (e.g., controlled design for interventions; cohort/case-control for exposures)?	√			
5	Were key potential confounders adequately controlled for (either in the design or statistical analysis)?	√			
6	Were the outcome/exposure measurements clearly defined, valid, and reliable (e.g., validated scales or objective measures)?	√			
7	Were the outcome/exposure measurements applied consistently across all participants?	√			
8	Was the sample size sufficiently powered to detect a statistically significant difference or association in the primary outcome?	√			
Domain 3: Data Completeness & Analysis (Information Bias)					
9	Were the data for key outcomes/exposures sufficiently complete (e.g., was attrition reported, <20%, or were missing data appropriately handled)?	√			
10	Were the statistical analyses appropriate (e.g., key assumptions tested, appropriate regression models used)?	√			
11	Were effect estimates (e.g., OR, RR, mean difference) with measures of precision (e.g., 95% CI) reported?	√			

Quantitative Study Quality Assessment (King et al., 2025)

	Assessment Criteria	Yes	No	Unclear	N/A
Domain 1: External Validity (Study Purpose & Population)					
1	Was the research question or objective clearly stated?	√			
2	Was the study population clearly defined and adequately described (e.g., inclusion/exclusion criteria)?	√			
3	Were the participants representative of the pre-specified target population?	√			
Domain 2: Internal Validity – Risk of Bias (Study Design & Execution)					
4	Was the study design appropriate for answering the research question (e.g., controlled design for interventions; cohort/case-control for exposures)?	√			
5	Were key potential confounders adequately controlled for (either in the design or statistical analysis)?	√			
6	Were the outcome/exposure measurements clearly defined, valid, and reliable (e.g., validated scales or objective measures)?	√			
7	Were the outcome/exposure measurements applied consistently across all participants?	√			
8	Was the sample size sufficiently powered to detect a statistically significant difference or association in the primary outcome?	√			
Domain 3: Data Completeness & Analysis (Information Bias)					
9	Were the data for key outcomes/exposures sufficiently complete (e.g., was attrition reported, <20%, or were missing data appropriately handled)?	√			
10	Were the statistical analyses appropriate (e.g., key assumptions tested, appropriate regression models used)?	√			
11	Were effect estimates (e.g., OR, RR, mean difference) with measures of precision (e.g., 95% CI) reported?	√			

Quantitative Study Quality Assessment (Romann et al., 2020)

	Assessment Criteria	Yes	No	Unclear	N/A
Domain 1: External Validity (Study Purpose & Population)					
1	Was the research question or objective clearly stated?	√			
2	Was the study population clearly defined and adequately described (e.g., inclusion/exclusion criteria)?	√			
3	Were the participants representative of the pre-specified target population?	√			
Domain 2: Internal Validity – Risk of Bias (Study Design & Execution)					
4	Was the study design appropriate for answering the research question (e.g., controlled design for interventions; cohort/case-control for exposures)?	√			
5	Were key potential confounders adequately controlled for (either in the design or statistical analysis)?			√	
6	Were the outcome/exposure measurements clearly defined, valid, and reliable (e.g., validated scales or objective measures)?	√			
7	Were the outcome/exposure measurements applied consistently across all participants?	√			
8	Was the sample size sufficiently powered to detect a statistically significant difference or association in the primary outcome?	√			
Domain 3: Data Completeness & Analysis (Information Bias)					
9	Were the data for key outcomes/exposures sufficiently complete (e.g., was attrition reported, <20%, or were missing data appropriately handled)?	√			
10	Were the statistical analyses appropriate (e.g., key assumptions tested, appropriate regression models used)?	√			
11	Were effect estimates (e.g., OR, RR, mean difference) with measures of precision (e.g., 95% CI) reported?	√			

Quantitative Study Quality Assessment (Towlson et al., 2021)

	Assessment Criteria	Yes	No	Unclear	N/A
Domain 1: External Validity (Study Purpose & Population)					
1	Was the research question or objective clearly stated?	√			
2	Was the study population clearly defined and adequately described (e.g., inclusion/exclusion criteria)?	√			
3	Were the participants representative of the pre-specified target population?	√			
Domain 2: Internal Validity – Risk of Bias (Study Design & Execution)					
4	Was the study design appropriate for answering the research question (e.g., controlled design for interventions; cohort/case-control for exposures)?	√			
5	Were key potential confounders adequately controlled for (either in the design or statistical analysis)?	√			
6	Were the outcome/exposure measurements clearly defined, valid, and reliable (e.g., validated scales or objective measures)?	√			
7	Were the outcome/exposure measurements applied consistently across all participants?	√			
8	Was the sample size sufficiently powered to detect a statistically significant difference or association in the primary outcome?	√			
Domain 3: Data Completeness & Analysis (Information Bias)					
9	Were the data for key outcomes/exposures sufficiently complete (e.g., was attrition reported, <20%, or were missing data appropriately handled)?	√			
10	Were the statistical analyses appropriate (e.g., key assumptions tested, appropriate regression models used)?	√			
11	Were effect estimates (e.g., OR, RR, mean difference) with measures of precision (e.g., 95% CI) reported?	√			

Quantitative Study Quality Assessment (Robinson et al., 2025)

	Assessment Criteria	Yes	No	Unclear	N/A
Domain 1: External Validity (Study Purpose & Population)					
1	Was the research question or objective clearly stated?	√			
2	Was the study population clearly defined and adequately described (e.g., inclusion/exclusion criteria)?	√			
3	Were the participants representative of the pre-specified target population?	√			
Domain 2: Internal Validity – Risk of Bias (Study Design & Execution)					
4	Was the study design appropriate for answering the research question (e.g., controlled design for interventions; cohort/case-control for exposures)?	√			
5	Were key potential confounders adequately controlled for (either in the design or statistical analysis)?	√			
6	Were the outcome/exposure measurements clearly defined, valid, and reliable (e.g., validated scales or objective measures)?	√			
7	Were the outcome/exposure measurements applied consistently across all participants?	√			
8	Was the sample size sufficiently powered to detect a statistically significant difference or association in the primary outcome?	√			
Domain 3: Data Completeness & Analysis (Information Bias)					
9	Were the data for key outcomes/exposures sufficiently complete (e.g., was attrition reported, <20%, or were missing data appropriately handled)?	√			
10	Were the statistical analyses appropriate (e.g., key assumptions tested, appropriate regression models used)?	√			
11	Were effect estimates (e.g., OR, RR, mean difference) with measures of precision (e.g., 95% CI) reported?	√			

Quantitative Study Quality Assessment (Arede et al., 2024)

	Assessment Criteria	Yes	No	Unclear	N/A
Domain 1: External Validity (Study Purpose & Population)					
1	Was the research question or objective clearly stated?	√			
2	Was the study population clearly defined and adequately described (e.g., inclusion/exclusion criteria)?	√			
3	Were the participants representative of the pre-specified target population?	√			
Domain 2: Internal Validity – Risk of Bias (Study Design & Execution)					
4	Was the study design appropriate for answering the research question (e.g., controlled design for interventions; cohort/case-control for exposures)?	√			
5	Were key potential confounders adequately controlled for (either in the design or statistical analysis)?	√			
6	Were the outcome/exposure measurements clearly defined, valid, and reliable (e.g., validated scales or objective measures)?	√			
7	Were the outcome/exposure measurements applied consistently across all participants?	√			
8	Was the sample size sufficiently powered to detect a statistically significant difference or association in the primary outcome?	√			
Domain 3: Data Completeness & Analysis (Information Bias)					
9	Were the data for key outcomes/exposures sufficiently complete (e.g., was attrition reported, <20%, or were missing data appropriately handled)?	√			
10	Were the statistical analyses appropriate (e.g., key assumptions tested, appropriate regression models used)?	√			
11	Were effect estimates (e.g., OR, RR, mean difference) with measures of precision (e.g., 95% CI) reported?	√			

Quantitative Study Quality Assessment (MacMaster et al., 2021)

	Assessment Criteria	Yes	No	Unclear	N/A
Domain 1: External Validity (Study Purpose & Population)					
1	Was the research question or objective clearly stated?	√			
2	Was the study population clearly defined and adequately described (e.g., inclusion/exclusion criteria)?	√			
3	Were the participants representative of the pre-specified target population?	√			
Domain 2: Internal Validity – Risk of Bias (Study Design & Execution)					
4	Was the study design appropriate for answering the research question (e.g., controlled design for interventions; cohort/case-control for exposures)?	√			
5	Were key potential confounders adequately controlled for (either in the design or statistical analysis)?	√			
6	Were the outcome/exposure measurements clearly defined, valid, and reliable (e.g., validated scales or objective measures)?	√			
7	Were the outcome/exposure measurements applied consistently across all participants?		√		
8	Was the sample size sufficiently powered to detect a statistically significant difference or association in the primary outcome?	√			
Domain 3: Data Completeness & Analysis (Information Bias)					
9	Were the data for key outcomes/exposures sufficiently complete (e.g., was attrition reported, <20%, or were missing data appropriately handled)?	√			
10	Were the statistical analyses appropriate (e.g., key assumptions tested, appropriate regression models used)?	√			
11	Were effect estimates (e.g., OR, RR, mean difference) with measures of precision (e.g., 95% CI) reported?	√			

Quantitative Study Quality Assessment (Segueida-Lorca et al., 2022)

	Assessment Criteria	Yes	No	Unclear	N/A
Domain 1: External Validity (Study Purpose & Population)					
1	Was the research question or objective clearly stated?	√			
2	Was the study population clearly defined and adequately described (e.g., inclusion/exclusion criteria)?	√			
3	Were the participants representative of the pre-specified target population?			√	
Domain 2: Internal Validity – Risk of Bias (Study Design & Execution)					
4	Was the study design appropriate for answering the research question (e.g., controlled design for interventions; cohort/case-control for exposures)?	√			
5	Were key potential confounders adequately controlled for (either in the design or statistical analysis)?	√			
6	Were the outcome/exposure measurements clearly defined, valid, and reliable (e.g., validated scales or objective measures)?	√			
7	Were the outcome/exposure measurements applied consistently across all participants?	√			
8	Was the sample size sufficiently powered to detect a statistically significant difference or association in the primary outcome?	√			
Domain 3: Data Completeness & Analysis (Information Bias)					
9	Were the data for key outcomes/exposures sufficiently complete (e.g., was attrition reported, <20%, or were missing data appropriately handled)?	√			
10	Were the statistical analyses appropriate (e.g., key assumptions tested, appropriate regression models used)?	√			
11	Were effect estimates (e.g., OR, RR, mean difference) with measures of precision (e.g., 95% CI) reported?	√			

Quantitative Study Quality Assessment (Salter et al., 2023)

	Assessment Criteria	Yes	No	Unclear	N/A
Domain 1: External Validity (Study Purpose & Population)					
1	Was the research question or objective clearly stated?	√			
2	Was the study population clearly defined and adequately described (e.g., inclusion/exclusion criteria)?	√			
3	Were the participants representative of the pre-specified target population?	√			
Domain 2: Internal Validity – Risk of Bias (Study Design & Execution)					
4	Was the study design appropriate for answering the research question (e.g., controlled design for interventions; cohort/case-control for exposures)?	√			
5	Were key potential confounders adequately controlled for (either in the design or statistical analysis)?	√			
6	Were the outcome/exposure measurements clearly defined, valid, and reliable (e.g., validated scales or objective measures)?	√			
7	Were the outcome/exposure measurements applied consistently across all participants?	√			
8	Was the sample size sufficiently powered to detect a statistically significant difference or association in the primary outcome?	√			
Domain 3: Data Completeness & Analysis (Information Bias)					
9	Were the data for key outcomes/exposures sufficiently complete (e.g., was attrition reported, <20%, or were missing data appropriately handled)?	√			
10	Were the statistical analyses appropriate (e.g., key assumptions tested, appropriate regression models used)?	√			
11	Were effect estimates (e.g., OR, RR, mean difference) with measures of precision (e.g., 95% CI) reported?	√			

Quantitative Study Quality Assessment (Barrett et al., 2022)

	Assessment Criteria	Yes	No	Unclear	N/A
Domain 1: External Validity (Study Purpose & Population)					
1	Was the research question or objective clearly stated?	√			
2	Was the study population clearly defined and adequately described (e.g., inclusion/exclusion criteria)?	√			
3	Were the participants representative of the pre-specified target population?	√			
Domain 2: Internal Validity – Risk of Bias (Study Design & Execution)					
4	Was the study design appropriate for answering the research question (e.g., controlled design for interventions; cohort/case-control for exposures)?	√			
5	Were key potential confounders adequately controlled for (either in the design or statistical analysis)?	√			
6	Were the outcome/exposure measurements clearly defined, valid, and reliable (e.g., validated scales or objective measures)?	√			
7	Were the outcome/exposure measurements applied consistently across all participants?	√			
8	Was the sample size sufficiently powered to detect a statistically significant difference or association in the primary outcome?			√	
Domain 3: Data Completeness & Analysis (Information Bias)					
9	Were the data for key outcomes/exposures sufficiently complete (e.g., was attrition reported, <20%, or were missing data appropriately handled)?	√			
10	Were the statistical analyses appropriate (e.g., key assumptions tested, appropriate regression models used)?	√			
11	Were effect estimates (e.g., OR, RR, mean difference) with measures of precision (e.g., 95% CI) reported?	√			

Qualitative Study Quality Assessment (Towlson & Cumming, 2022)

	Assessment Criteria	Yes	No	Unclear	N/A
Domain 1: Philosophical & Methodological Congruence					
1	Is there congruity between the stated philosophical perspective and the research methodology?			√	
2	Is there congruity between the research methodology and the research question or objectives?	√			
3	Is there congruity between the research methodology and the methods used to collect data?			√	
4	Is there congruity between the research methodology and the representation and analysis of data?	√			
5	Is there congruity between the research methodology and the interpretation of results	√			
Domain 2: Researcher Reflexivity & Participant Representation					
6	Is there a statement locating the researcher culturally or theoretically (i.e., researcher positionality)?	√			
7	Is the influence of the researcher on the research, and vice-versa, adequately addressed?	√			
8	Are participants, and their voices, adequately represented (e.g., supported by direct quotes)?	√			
Domain 3: Ethical Rigor & Analytical Trustworthiness					
9	Is the research ethical according to current criteria, and is there evidence of ethical approval by an appropriate body?	√			
10	Are the findings adequately derived from the data (e.g., clear thematic development)?			√	
11	Do the conclusions drawn in the research report flow logically from the analysis and interpretation of the data?	√			

Qualitative Study Quality Assessment (Cumming et al., 2018)

	Assessment Criteria	Yes	No	Unclear	N/A
Domain 1: Philosophical & Methodological Congruence					
1	Is there congruity between the stated philosophical perspective and the research methodology?			√	
2	Is there congruity between the research methodology and the research question or objectives?	√			
3	Is there congruity between the research methodology and the methods used to collect data?	√			
4	Is there congruity between the research methodology and the representation and analysis of data?	√			
5	Is there congruity between the research methodology and the interpretation of results	√			
Domain 2: Researcher Reflexivity & Participant Representation					
6	Is there a statement locating the researcher culturally or theoretically (i.e., researcher positionality)?			√	
7	Is the influence of the researcher on the research, and vice-versa, adequately addressed?			√	
8	Are participants, and their voices, adequately represented (e.g., supported by direct quotes)?	√			
Domain 3: Ethical Rigor & Analytical Trustworthiness					
9	Is the research ethical according to current criteria, and is there evidence of ethical approval by an appropriate body?	√			
10	Are the findings adequately derived from the data (e.g., clear thematic development)?	√			
11	Do the conclusions drawn in the research report flow logically from the analysis and interpretation of the data?	√			

Qualitative Study Quality Assessment (Arroyo Moya, 2023)

	Assessment Criteria	Yes	No	Unclear	N/A
Domain 1: Philosophical & Methodological Congruence					
1	Is there congruity between the stated philosophical perspective and the research methodology?			√	
2	Is there congruity between the research methodology and the research question or objectives?	√			
3	Is there congruity between the research methodology and the methods used to collect data?	√			
4	Is there congruity between the research methodology and the representation and analysis of data?	√			
5	Is there congruity between the research methodology and the interpretation of results	√			
Domain 2: Researcher Reflexivity & Participant Representation					
6	Is there a statement locating the researcher culturally or theoretically (i.e., researcher positionality)?	√			
7	Is the influence of the researcher on the research, and vice-versa, adequately addressed?	√			
8	Are participants, and their voices, adequately represented (e.g., supported by direct quotes)?	√			
Domain 3: Ethical Rigor & Analytical Trustworthiness					
9	Is the research ethical according to current criteria, and is there evidence of ethical approval by an appropriate body?	√			
10	Are the findings adequately derived from the data (e.g., clear thematic development)?	√			
11	Do the conclusions drawn in the research report flow logically from the analysis and interpretation of the data?	√			

Qualitative Study Quality Assessment (Reeves et al., 2018)

	Assessment Criteria	Yes	No	Unclear	N/A
Domain 1: Philosophical & Methodological Congruence					
1	Is there congruity between the stated philosophical perspective and the research methodology?			√	
2	Is there congruity between the research methodology and the research question or objectives?	√			
3	Is there congruity between the research methodology and the methods used to collect data?	√			
4	Is there congruity between the research methodology and the representation and analysis of data?	√			
5	Is there congruity between the research methodology and the interpretation of results	√			
Domain 2: Researcher Reflexivity & Participant Representation					
6	Is there a statement locating the researcher culturally or theoretically (i.e., researcher positionality)?			√	
7	Is the influence of the researcher on the research, and vice-versa, adequately addressed?			√	
8	Are participants, and their voices, adequately represented (e.g., supported by direct quotes)?	√			
Domain 3: Ethical Rigor & Analytical Trustworthiness					
9	Is the research ethical according to current criteria, and is there evidence of ethical approval by an appropriate body?	√			
10	Are the findings adequately derived from the data (e.g., clear thematic development)?	√			
11	Do the conclusions drawn in the research report flow logically from the analysis and interpretation of the data?	√			

Mixed Methods Study Quality Assessment (Towlson et al., 2023)

	Assessment Criteria	Yes	No	Unclear	N/A
Domain 1: Overall Design Rationale & Integration					
1	Is there an adequate rationale for using a mixed methods design to address the research question?	√			
2	Is the temporal sequence of the quantitative and qualitative components clearly specified (e.g., concurrent or sequential)?	√			
3	Are the different components of the study effectively integrated to answer the research question?	√			
Domain 2: Interpretation & Handling of Divergence					
4	Are the outputs of the integration of qualitative and quantitative components adequately interpreted?	√			
5	Are divergences and inconsistencies between quantitative and qualitative results adequately addressed?			√	
Domain 3: Component-Level Quality					
6	Does the quantitative component adhere to the quality criteria appropriate for its design (e.g., RCT, non-randomized, or descriptive)?	√			
7	Does the qualitative component adhere to the quality criteria appropriate for its design (e.g., phenomenology, grounded theory)?			√	

Mixed Methods Study Quality Assessment (Bradley et al., 2019)

	Assessment Criteria	Yes	No	Unclear	N/A
Domain 1: Overall Design Rationale & Integration					
1	Is there an adequate rationale for	√			

	using a mixed methods design to address the research question?			
2	Is the temporal sequence of the quantitative and qualitative components clearly specified (e.g., concurrent or sequential)?	√		
3	Are the different components of the study effectively integrated to answer the research question?	√		
Domain 2: Interpretation & Handling of Divergence				
4	Are the outputs of the integration of qualitative and quantitative components adequately interpreted?	√		
5	Are divergences and inconsistencies between quantitative and qualitative results adequately addressed?	√		
Domain 3: Component-Level Quality				
6	Does the quantitative component adhere to the quality criteria appropriate for its design (e.g., RCT, non-randomized, or descriptive)?	√		
7	Does the qualitative component adhere to the quality criteria appropriate for its design (e.g., phenomenology, grounded theory)?			√
